# Role of Extracellular DNA in Dalbavancin Activity against Methicillin-Resistant Staphylococcus aureus (MRSA) Biofilms in Patients with Skin and Soft Tissue Infections

**DOI:** 10.1128/spectrum.00351-22

**Published:** 2022-04-13

**Authors:** Francesca Sivori, Ilaria Cavallo, Daniela Kovacs, Maria Guembe, Isabella Sperduti, Mauro Truglio, Martina Pasqua, Grazia Prignano, Arianna Mastrofrancesco, Luigi Toma, Fulvia Pimpinelli, Aldo Morrone, Fabrizio Ensoli, Enea Gino Di Domenico

**Affiliations:** a Microbiology and Virology, IRCCS San Gallicano Dermatological Institute, Rome, Italy; b Cutaneous Physiopathology, San Gallicano Dermatological Institute, IRCCS, Rome, Italy; c Department of Clinical Microbiology and Infectious Diseases, Hospital General Universitario Gregorio Marañón, Instituto de Investigación Sanitaria Gregorio Marañón, Madrid, Spain; d Biostatistical Unit-Clinical Trials Center, IRCCS Regina Elena National Cancer Institute, Rome, Italy; e Department of Biology and Biotechnology “C. Darwin”, Sapienza University, Rome, Italy; f Department of Research, Advanced Diagnostics, and Technological Innovation, Translational Research Area, IRCCS Regina Elena National Cancer Institute, Rome, Italy; g Scientific Direction, IRCCS San Gallicano Dermatological Institute, Rome, Italy; Forschungszentrum Jülich GmbH

**Keywords:** biofilm, dalbavancin, *Staphylococcus aureus*, MRSA, extracellular DNA

## Abstract

Methicillin-resistant Staphylococcus aureus (MRSA) has become the leading cause of skin and soft tissue infections (SSTIs). Biofilm production further complicates patient treatment, contributing to increased bacterial persistence and antibiotic tolerance. The study aimed to explore the efficacy of different antibiotics on biofilm-producing MRSA isolated from patients with SSTI. A total of 32 MRSA strains were collected from patients with SSTI. The MIC and minimal biofilm eradication concentration (MBEC) were measured in planktonic and biofilm growth. The study showed that dalbavancin, linezolid, and vancomycin all inhibited MRSA growth at their EUCAST susceptible breakpoint. Of the MRSA strains, 87.5% (*n* = 28) were strong biofilm producers (SBPs), while only 12.5% (*n* = 4) were weak biofilm producers (WBPs). The MBEC_90_ values for dalbavancin were significantly lower than those of linezolid and vancomycin in all tested strains. We also found that extracellular DNA (eDNA) contributes to the initial microbial attachment and biofilm formation. The amount of eDNA differed among MRSA strains and was significantly higher in those isolates with high dalbavancin and vancomycin tolerance. Exogenously added DNA increased the MBEC_90_ and protection of biofilm cells from dalbavancin activity. Of note, the relative abundance of eDNA was higher in MRSA biofilms exposed to MBEC_90_ dalbavancin than in untreated MRSA biofilms and those exposed to sub-MIC_90_. Overall, dalbavancin was the most active antibiotic against MRSA biofilms at concentrations achievable in the human serum. Moreover, the evidence of a drug-related increase of eDNA and its contribution to antimicrobial drug tolerance reveals novel potential targets for antibiofilm strategies against MRSA.

**IMPORTANCE** Staphylococcus aureus is the most common cause of skin and soft tissue infections (SSTIs) worldwide. In addition, methicillin-resistant S. aureus (MRSA) is increasingly frequent in postoperative infections and responsible for a large number of hospital readmissions and deaths. Biofilm formation by S. aureus is a primary risk factor in SSTIs, due to a higher antibiotic tolerance. Our study showed that the biofilm-forming capacity varied among MRSA strains, although strong biofilm producers were significantly more abundant than weak biofilm producer strains. Notably, dalbavancin demonstrated a potent antibiofilm activity at concentrations achievable in human serum. Nevertheless, dalbavancin activity was affected by an increased concentration of extracellular DNA in the biofilm matrix. This study provides novel insight for designing more targeted therapeutic strategies against MRSA and to prevent or eradicate harmful biofilms.

## INTRODUCTION

Skin and soft tissue infections (SSTIs) represent severe forms of infectious diseases that involve deeper soft tissues, responsible for significant risk of relapse, prolonged hospitalization, and death (1, 2). Although many hospitals have adopted specific measures to reduce the emergence of adverse events and increase the effectiveness of surgical procedures, SSTIs remain a challenging and costly problem (1, 2). Staphylococcus aureus is the most common cause of SSTIs worldwide. In addition, methicillin-resistant S. aureus (MRSA) is increasingly frequent in postoperative infection and responsible for a significant increase in the risk of death and hospital readmission compared to uninfected surgical patients (1, 3–8). Biofilm represents an additional risk factor in SSTIs, primarily due to a higher antibiotic tolerance (9–11). Indeed, biofilm structure hinders antibiotic penetration, depending on the matrix composition and structure.

In particular, S. aureus biofilm matrix is mainly composed of polysaccharides, extracellular DNA (eDNA), and proteins (12). S. aureus biofilm production is achieved by two major pathways, alternatively leading to the assembly of a polysaccharide-based or an eDNA/protein biofilm. Although these distinctive forms of biofilms are not mutually exclusive, the polysaccharide-based biofilm is predominantly observed in methicillin-sensitive S. aureus (MSSA) strains, while the eDNA/protein biofilm is more represented in MRSA strains (13–16).

Biofilm eradication is critical for the effective treatment of SSTIs; however, no specific drugs are available as yet. Dalbavancin is a novel lipoglycopeptide with a half-life of 14.4 days and is approved for the single-dose treatment of acute bacterial SSTIs in adults (17). Like other glycopeptides, dalbavancin inhibits cell wall peptidoglycan cross-linking, showing activity against certain vancomycin-resistant enterococci (VanB and VanC phenotype) (17). Previous data indicate that dalbavancin has a remarkable efficacy against MRSA, thus representing a promising antimicrobial agent against staphylococcal biofilms (18–21). Currently, no data are available on the efficacy of dalbavancin in the treatment of biofilm-growing MRSA from SSTIs. The present study explores the activity of dalbavancin against planktonic and biofilm-growing MRSA isolated from patients with SSTIs. Furthermore, we investigated the potential impact of eDNA on microbial drug tolerance and dalbavancin efficacy for future, more targeted eradication strategies.

## RESULTS

From January 2018 to November 2020, 32 MRSA strains were isolated from patients presenting with SSTIs (Table 1). The sites of isolation were surgical wound (*n* = 13; 40.6%), ulcer (*n* = 9; 28.2%), abscess (*n* = 5; 15.6%), cellulitis (*n* = 4; 12.5%) and necrotizing fasciitis (*n* = 1; 3.1%).

**TABLE 1:** Demographic and clinical features of patients at enrollment (*n* = 32)^*a*^

Characteristic or site	Value	Percent
Demographic characteristics		
Sex (no. female)	13	40.6
Age, yrs (range)	58.9 (41.5–79.1)	
Site of isolation (no.)		
Surgical wound	13	40.6
Ulcer	9	28.2
Abscess	5	15.6
Cellulitis	4	12.5
Necrotizing fasciitis	1	3.1

a Age is expressed as the mean, with the range in parentheses.

**FIG 1:**
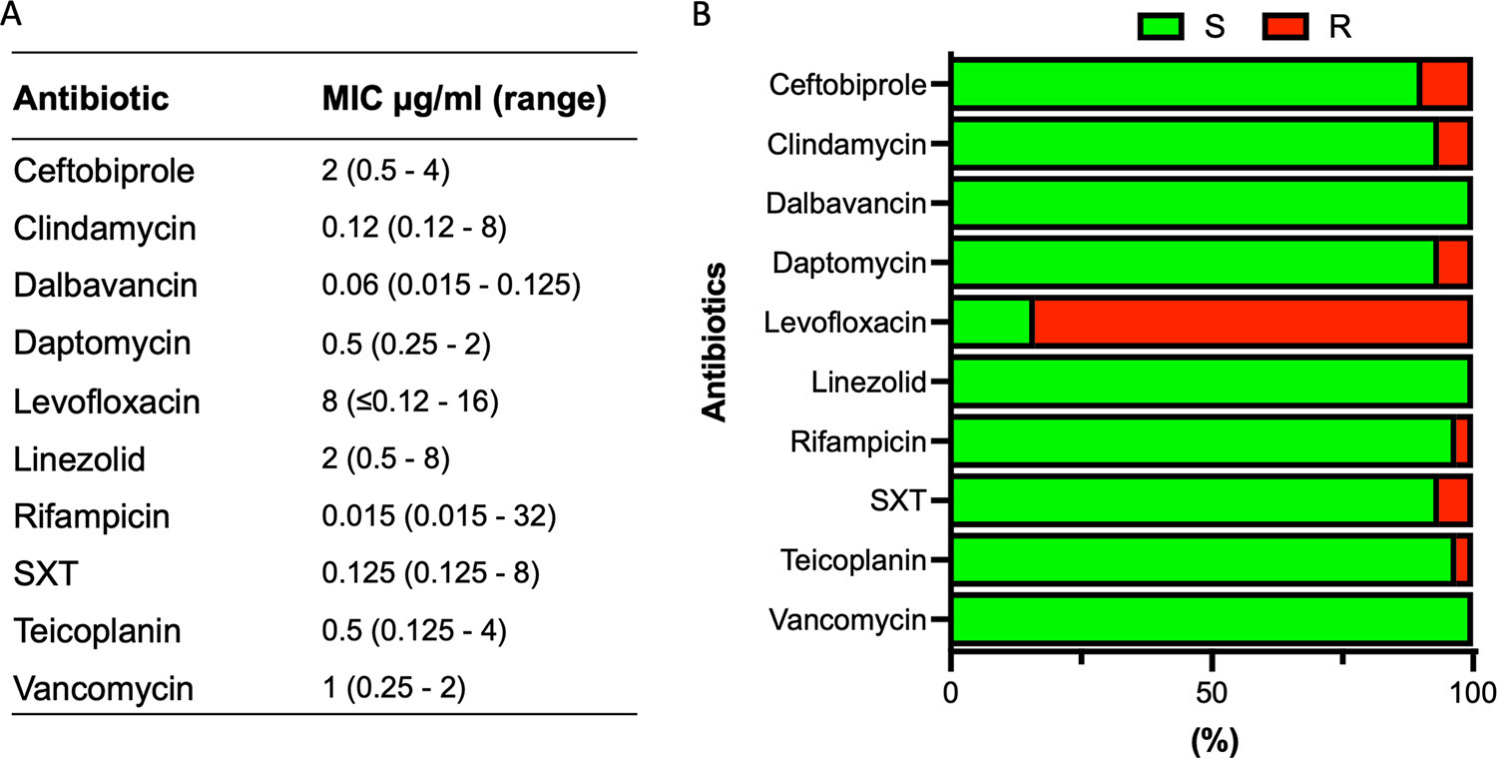
Antibiotic susceptibility profile of 32 methicillin-resistant Staphylococcus aureus (MRSA) strains to the indicated antimicrobials as determined by broth microdilution test. (A) Median MIC. (B) Percentage of susceptible (green) and resistant (red) strains to the indicated antimicrobials. Classification was performed according to the European Committee on Antimicrobial Susceptibility Testing clinical breakpoint tables (EUCAST clinical breakpoint). SXT, trimethoprim/sulfamethoxazole.

The MIC of conventional antibiotics against MRSA isolates are summarized in Fig. 1. Levofloxacin showed the highest level of antimicrobial resistance (83.9%; MIC = 8 μg/mL, range < 0.12 to 8). Conversely, MRSA strains were totally susceptible to dalbavancin (MIC = 0.06 μg/mL, range = 0.015 to 0.125), linezolid (MIC = 2 μg/mL, range = 0.5 to 8 μg/mL), and vancomycin (MIC = 1μg/mL, range = 0.25 to 2).

**FIG 2:**
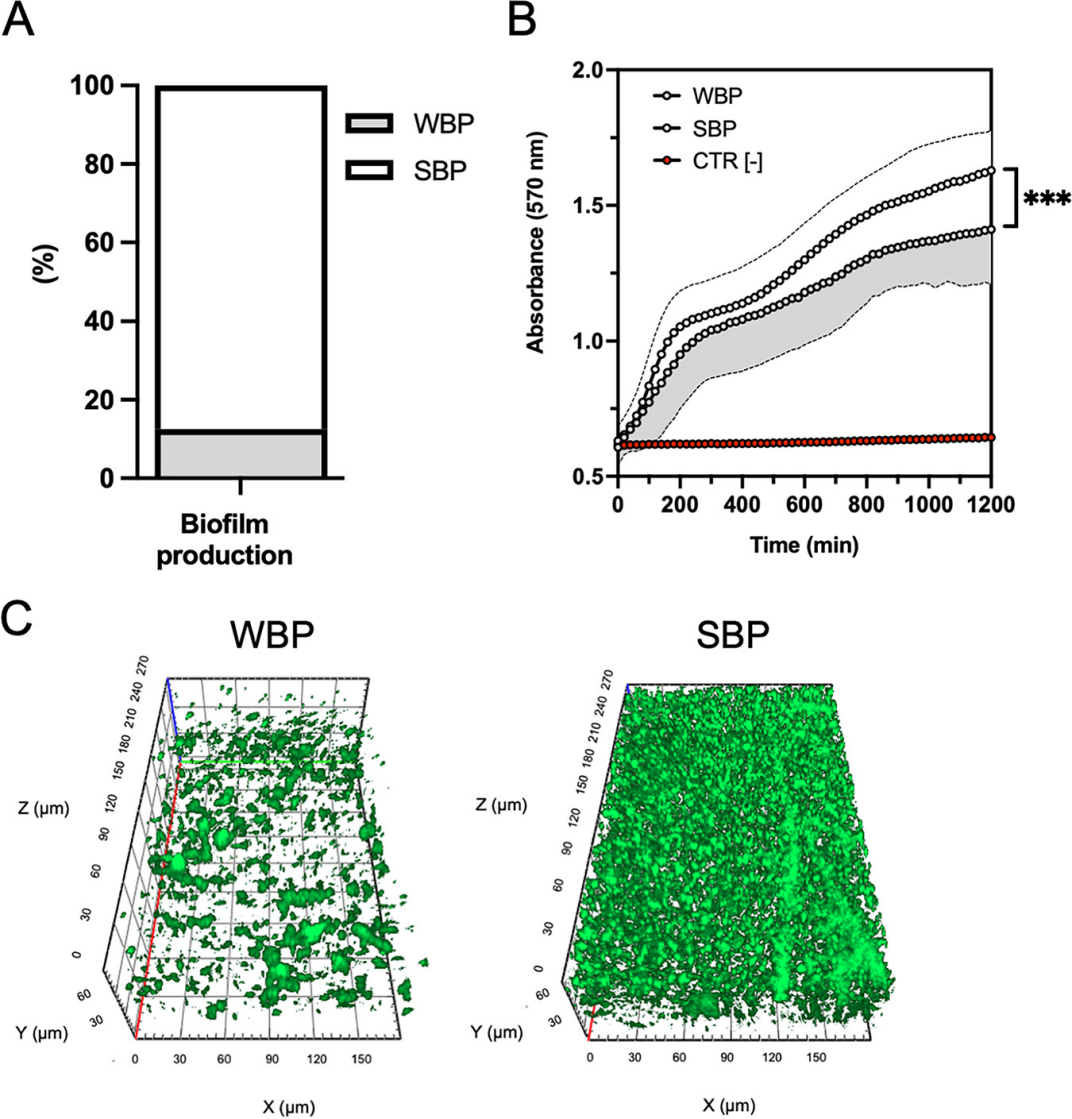
(A) Biofilm formation measured by the clinical BioFilm Ring Test (cBRT) for MRSA strains. Clinical isolates were classified as weak biofilm producers (WBP) and strong biofilm producers (SBP). All results were expressed as a percentage of strains with the specific biofilm-forming ability. (B) Resazurin conversion into resorufin measured after 20 h of incubation, for WBP and SBP. ***, *P* < 0.001, using the Mann-Whitney test for the absorbance values after 1,200 min. Data represent means and the standard errors of two independent experiments analyzed in duplicate. (C) Representative Apotome microscopy images of WBP (left panel) and SBP (right panel) MRSA biofilms, stained with the Live/Dead BacLight bacterial viability kit and analyzed with ZEN 3.1 software, after 20 h of incubation at 37°C. Orthogonal sections displaying horizontal (*z*) and side views (*x* and *y*) of reconstructed 3-dimensional (3D) biofilm images are shown.

The assessment of biofilm formation showed that strong biofilm-producers (SBPs) (*n* = 28, 87.5%) were significantly (*P* < 0.001) more abundant than weak biofilm-producers (WBPs) (*n* = 4, 12.5%) among MRSA isolates (Fig. 2A). The resazurin conversion into resorufin revealed that the strains classified as WBPs showed a significantly (*P* < 0.001) lower level of resazurin reduction (absorbance at 570 nm = 1.62 ± 0.15) than that of SBPs (Fig. 2B). Apotome microscopy analysis of the biofilms examined after 20 h of incubation showed that all the SBPs gave a full coverage throughout the extension of the substrate with the development of uniform and thick biofilms of 25 to 60 μm in height, while WBPs achieved a partial coverage of the substrate with the development of uneven biofilms of 10 to 30 μm in height (Fig. 2C).

**FIG 3:**
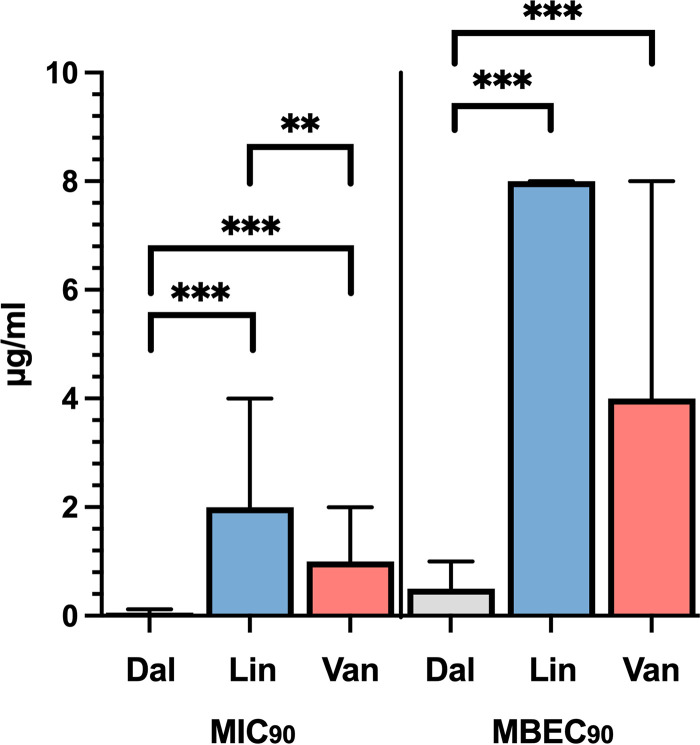
Dalbavancin showed *in vitro* activity against MRSA biofilms. Median (range) MIC_90_ and MBEC_90_ (minimum biofilm eradication concentration) for dalbavancin (Dal), linezolid (Lin), and vancomycin (Van) determined for MRSA strains. Statistical differences were determined using the Kruskal-Wallis test followed by Dunn’s *post hoc* test for multiple comparisons; *, *P* < 0.05; **, *P* < 0.01; ***, *P* < 0.001.

Most of the MRSA strains were classified as SBP. Thus, we evaluated whether dalbavancin, linezolid, and vancomycin, the most effective antibiotics against planktonic MRSA, were active against all MRSA strains in the biofilm phase. To this end, we compared the differences in MIC_90_ and minimal biofilm eradication concentration at which 90% of the tested isolates are inhibited (MBEC_90_) (Fig. 3). The MIC_90_ was 0.06 μg/mL (range, 0.015 to 0.125 μg/mL) for dalbavancin, 2 μg/mL (range, 0.5 to 4 μg/mL) for linezolid, and 1 μg/mL (range, 0.25 to 2 μg/mL) for vancomycin. The MIC_90_ for dalbavancin was significantly lower than those for linezolid (*P* < 0.001) and vancomycin (*P* < 0.001). In additions, the MIC_90_ for linezolid was found to be significantly (*P* < 0.001) higher than that for vancomycin. Next, dalbavancin, linezolid, and vancomycin activity was assessed against MRSA biofilms. The MBEC_90_ was 0.5 μg/mL (range, 0.12 to 0.5 μg/mL) for dalbavancin, 8 μg/mL (range, 2 to 8 μg/mL) for linezolid, and 4 μg/mL (range, 2 to 8 μg/mL) for vancomycin. Notably, dalbavancin showed *in vitro* activity against MRSA biofilms with MBEC_90_ values significantly lower than those of linezolid (*P* < 0.001) and vancomycin (*P* < 0.001) (Fig. 3). No significant difference in the MBEC_90_ values was observed between linezolid and vancomycin.

**FIG 4:**
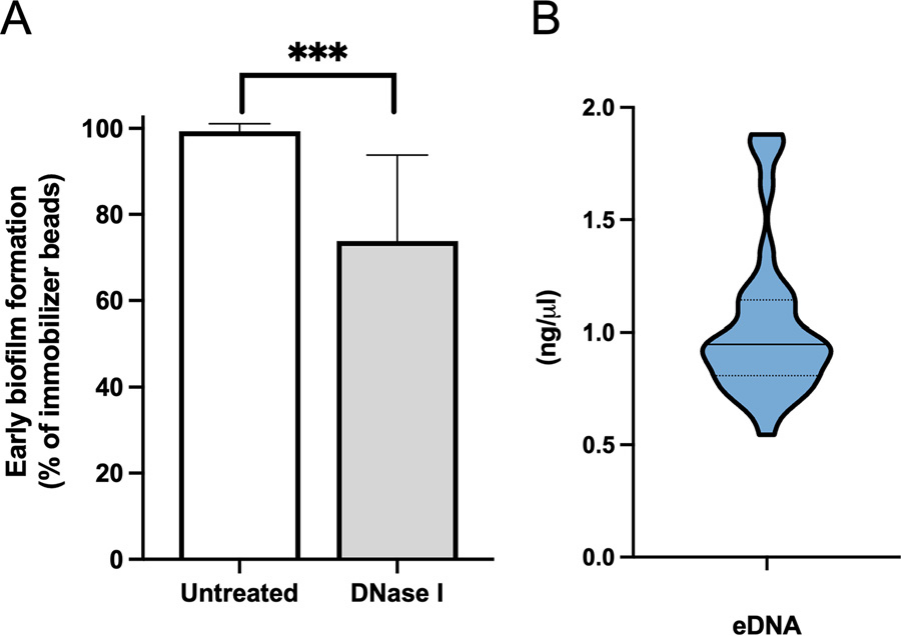
DNase I reduces MRSA biofilm. (A) Role of extracellular DNA (eDNA) on early biofilm formation for MRSA strains. Results are expressed as relative differences (equation 1) in the amounts of biofilm as measured by the BioFilm Ring Test after 6 h of incubation in the presence of DNase compared with untreated control strains. Data represent means and the corresponding standard errors of two independent experiments analyzed in duplicate. ***, *P* < 0.001, using the Mann-Whitney test. (B) Quantification of eDNA in MRSA biofilm cultures. Black bars and dots represent medians and quartiles.

The attachment and initiation of biofilm formation were investigated by measuring the relative difference (RD) (equation 1 in Materials and Methods) of bead immobilizations in the presence and absence of DNase I. The results showed that the treatment with DNase I leads to a significant (*P* < 0.001) reduction of 26.0 ± 20.4% in the initial attachment of MRSA strains compared to untreated isolates (Fig. 4A). Notably, eDNA was detected in all biofilm samples at a median concentration of 0.95 ng/μL (95% confidence interval [CI], 0.82 to 1.08) (Fig. 4B). However, the eDNA concentration in the biofilm did not correlate with the level of biofilm production measured by the clinical BioFilm Ring Test (cBRT).

**FIG 5:**
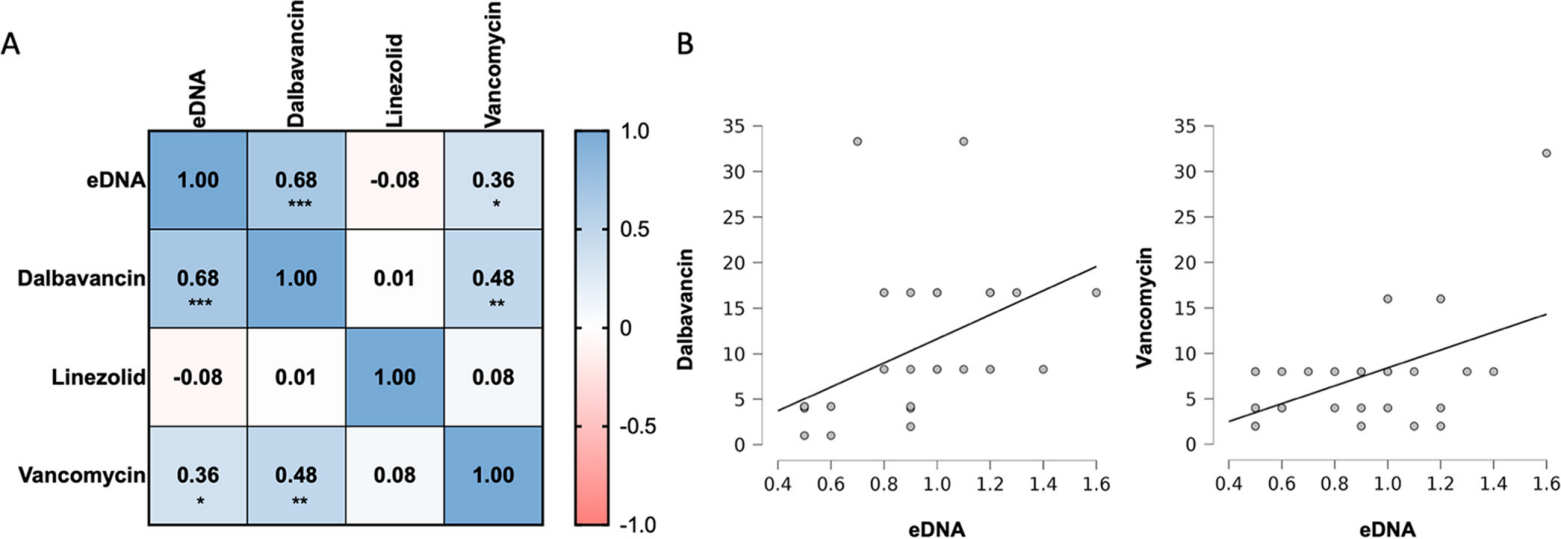
(A) Correlation matrix based on Spearman’s rank correlation coefficient between the level of eDNA and the biofilm tolerance (BT) for dalbavancin, linezolid, and vancomycin. The BT is the ratio between the MBEC_90_ (minimum biofilm eradication concentration) and MIC_90_ values calculated for each antimicrobial agent and strain. (B) Scatter graphs with detailed illustration of the relation between eDNA level and BT for dalbavancin and vancomycin.

It has been reported that eDNA can contribute to antibiotic tolerance in MRSA (22). Thus, the ratio between MBEC_90_ and MIC_90_ was used to quantify the biofilm tolerance (BT) score to dalbavancin, linezolid, and vancomycin. We calculated the correlation coefficient between the eDNA levels and BT to dalbavancin, linezolid, and vancomycin (Fig. 5). The correlation matrix revealed a significant correlation of eDNA with the BT for dalbavancin (ρ = 0.68; *P* < 0.001) and a positive correlation with vancomycin (ρ = 0.36; *P* = 0.04). Thus, MRSA strains were more likely to present an increase in BT for dalbavancin and vancomycin when the level of eDNA was high. In particular, the glycopeptide antibiotics dalbavancin and vancomycin also revealed a positive correlation (ρ = 0.48; *P* = 0.005), suggesting the presence of a common mechanism for BT likely based on the level of eDNA in the biofilm matrix. Conversely, no correlation was observed between BT to linezolid and the levels of eDNA.

**FIG 6:**
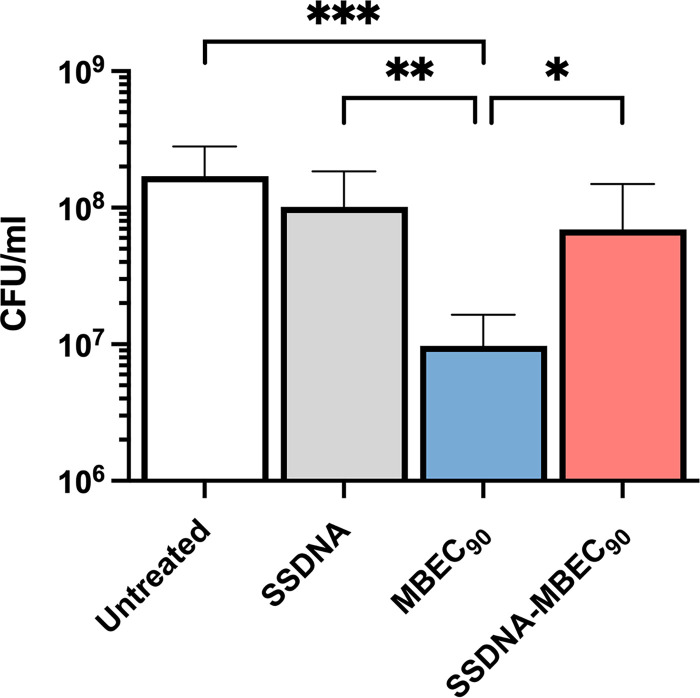
Exogenous DNA protects biofilm cells from dalbavancin. Number of viable cells (CFU/mL) obtained from 20-h MRSA biofilms treated with MBEC_90_ of dalbavancin, 16 μg/mL of exogenous salmon sperm DNA, and a solution of salmon sperm DNA and dalbavancin. All biofilms were exposed to the treatments for 20 h. Statistical differences were determined using the Kruskal-Wallis test followed by Dunn’s *post hoc* test for multiple comparisons; *, *P* < 0.05; **, *P* < 0.01; ***, *P* < 0.001.

Next, MRSA biofilms were exposed to the MBEC_90_ of dalbavancin in the presence or in the absence of salmon sperm DNA to verify whether the addition of exogenous DNA could protect biofilm cells. Data showed that exogenous DNA alone did not affect the growth of biofilm cells. As expected, dalbavancin at the MBEC_90_ concentration caused a significant (*P* < 0.001) reduction in the number of biofilm cells compared to untreated biofilm cells (Fig. 6). Notably, preincubation of dalbavancin at a concentration of MBEC_90_ with salmon sperm DNA significantly (*P* = 0.011) increases the antimicrobial tolerance of biofilm cells compared to dalbavancin alone.

**FIG 7:**
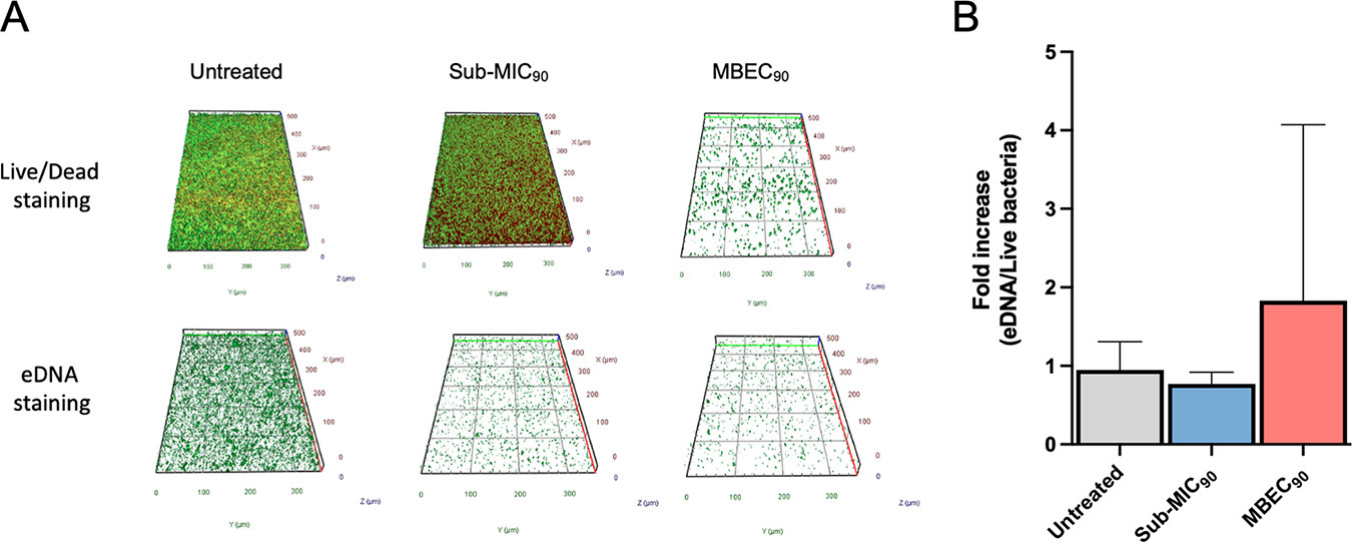
(A) Representative images of biofilm cells stained with the Live/Dead BacLight bacterial viability kit (BacLight kit) and TOTO-1 for eDNA (green) exposed to dalbavancin (sub-MIC_90_ and MBEC_90_) compared to untreated biofilms. (B) Relative abundances of fluorescence intensity of eDNA stained by TOTO-1 and live bacteria stained with the Live/Dead BacLight bacterial viability kit, analyzed with AxioVision 4.8 software, and expressed as the fold increase compared to untreated control cells.

**FIG 8:**
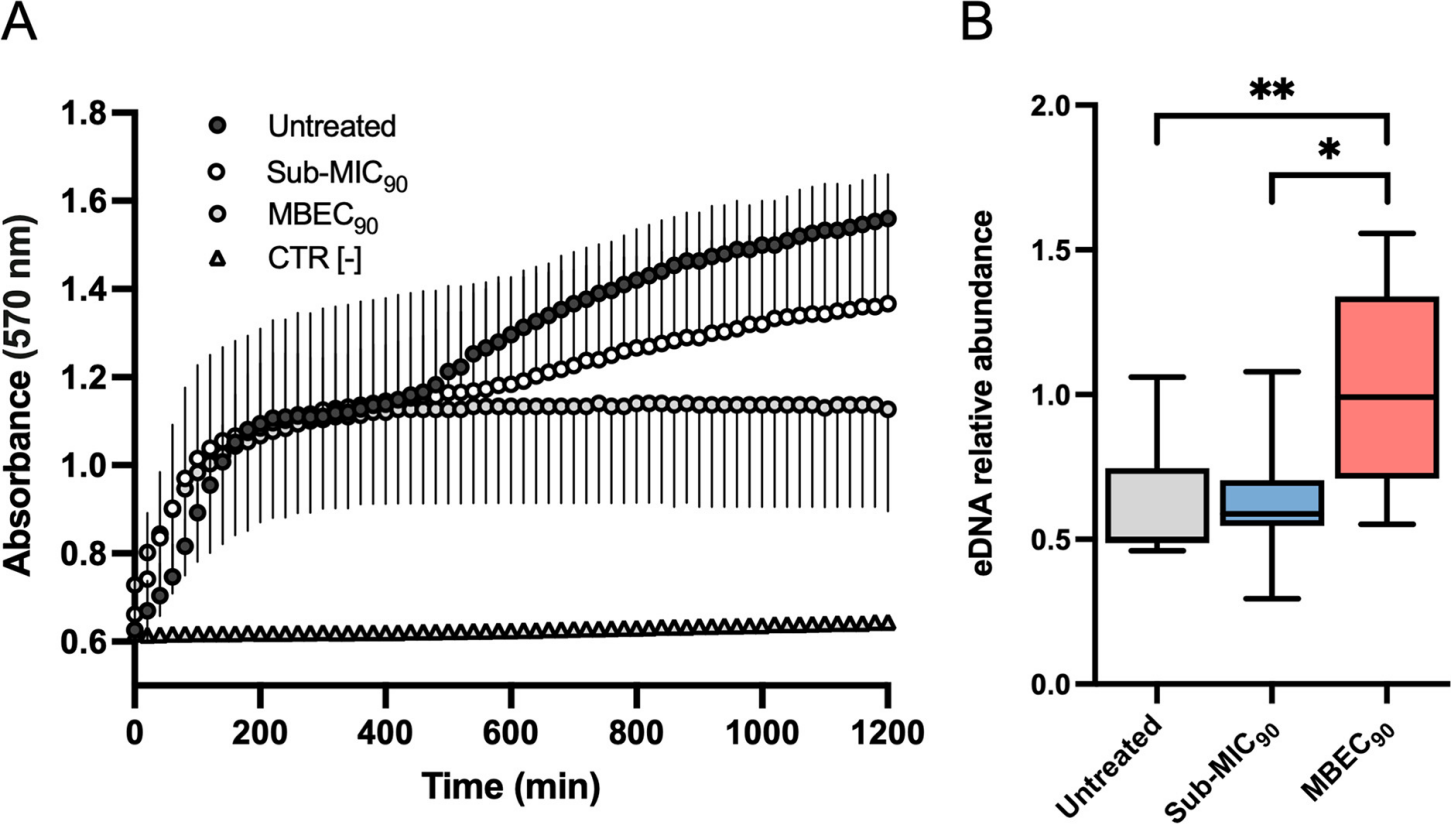
Increased relative abundance of eDNA in MRSA biofilms exposed to dalbavancin at MBEC_90_. (A) Biofilm viability (resazurin conversion to resorufin) at 20 h in untreated and treated at sub-MIC_90_ and MBEC_90_ MRSA strains. (B) eDNA relative abundances normalized to biofilm viability at absorbance at 570 nm. Statistical differences were determined using the Kruskal-Wallis test followed by Dunn’s *post hoc* test for multiple comparisons; *, *P* < 0.05; **, *P* < 0.01; ***, *P* < 0.001.

Previous work suggested that staphylococcal biofilms treated with antibiotics at sub-MIC levels contained more eDNA than untreated controls (22, 23). To this end, eDNA was analyzed by Apotome microscopy in 20-h-old MRSA biofilms exposed to sub-MIC and MBEC_90_ levels of dalbavancin (Fig. 7A). Distinct differences in the biofilm structure and thickness were observed between untreated biofilms and those treated with the sub-MIC_90_ and MBEC_90_. The presence of eDNA was determined by staining with TOTO-1, which is impermeable to live bacterial cells and normalized to the living cells in the biofilms as measured by the Live/Dead assay. The relative amount of eDNA in MRSA biofilms treated with dalbavancin at sub-MIC_90_ levels was comparable to that in untreated biofilms (Fig. 7B). Conversely, MRSA biofilms treated with dalbavancin at the MBEC_90_ showed a 2-fold increase in the relative abundance of eDNA than untreated controls and sub-MIC_90_-treated strains. The relative amount of eDNA normalized to biofilm viability was calculated to confirm these observations. MRSA biofilms treated with dalbavancin at sub-MIC_90_ levels contained a relative abundance of eDNA comparable to that of untreated biofilms (*P* > 0.05) (Fig. 8A). Conversely, the relative abundance of eDNA in MRSA biofilms treated with dalbavancin at the MBEC_90_ was significantly higher than that of untreated control strains (*P* = 0.004) and those treated with sub-MIC_90_ (*P* = 0.026) concentrations (Fig. 8B).

## DISCUSSION

Data from this study are consistent with previous reports showing that dalbavancin, linezolid, and vancomycin are effective in managing SSTIs due to MRSA and further confirm that biofilm poses a significant challenge for effective treatment of SSTI (24–28). Biofilm formation by S. aureus is associated with a delay in reepithelialization of the infected tissues, ultimately increasing healing time (29–34). Our study showed that the biofilm-forming capacity varied among MRSA strains, although SBPs (87.5%) were significantly more abundant than WBPs (12.5%). Biofilm production analyzed by Apotome microscopy showed that all the SBPs gave full coverage throughout the substrate extension, developing uniform and thick biofilms of 25 to 60 μm in height. Conversely, biofilm biomass was strongly reduced in WBPs, reaching only limited substrate coverage and the development of uneven biofilms of 10 to 30 μm in height. These results are consistent with previous studies reporting a high percentage (70 to 95%) of biofilm-forming S. aureus isolates from SSTI, confirming that surface adhesion is essential for skin colonization and infection (34–39). Due to the high percentage of SBP isolates, the activity of dalbavancin, linezolid, and vancomycin, which were the most effective antibiotics on planktonic cells according to the MIC values, was evaluated against MRSA biofilm. Dalbavancin showed potent activity against established MRSA biofilms, with an MBEC_90_ of 0.5 μg/mL (range, 0.12 to 0.5 μg/mL). The antibiofilm activity of dalbavancin was superior to that of the other drugs, with an MBEC_90_ significantly lower than that of linezolid (8 μg/mL; range, 2 to 8 μg/mL) and vancomycin (4 μg/mL; range, 2 to 8 μg/mL), which are among the most common antibiotics administered for the treatment of biofilm-related infections caused by MRSA (40). Notably, the study showed that dalbavancin was active *in vitro* against MRSA biofilms in concentrations achievable in human serum, as the mean plasma concentration is >30 mg/L for 7 days after one dose of 1,000 mg (17). Previous studies have shown promising activity of dalbavancin against Gram-positive biofilms, including MRSA (20, 41–45). Moreover, an animal study model reported that dalbavancin was more effective than vancomycin in preventing S. aureus colonization of medical devices (44). In rat sternal osteomyelitis, dalbavancin was active in the treatment of MRSA (46). Interestingly, although dalbavancin failed to eradicate MRSA biofilms from a foreign-body infection model, it was able to eradicate biofilms when used in combination with rifampicin, achieving cure rates of 25 to 36% compared to monotherapy and preventing the rifampicin resistance (18). It has been suggested that the activity of dalbavancin can be facilitated by its mechanism of action. Indeed, this antibiotic not only inhibits bacterial cell wall synthesis like vancomycin but also can dimerize and anchor its lipophilic side chain in the bacterial membranes (47, 48). This may increase the affinity of dalbavancin for its target, improving antimicrobial potency. In addition, previous data have reported that vancomycin does not efficiently penetrate staphylococcal biofilms, suggesting that dalbavancin may better diffuse and preserve its activity against biofilm-embedded cells (19, 22, 49, 50). Unlike glycopeptides, linezolid has a different mechanism of action based on protein synthesis inhibition (51). Our findings are in line with previous studies indicating that linezolid is poorly effective in eradicating established MRSA biofilms (52, 53). Others found a decrease in bacterial counts in the presence of linezolid but could not demonstrate biofilm eradication (52–56). A previous meta-analysis showed that linezolid is more effective than vancomycin for treating people with SSTIs, including those caused by MRSA. However, the authors warned of the potential risk of bias due to several studies supported by the pharmaceutical company that produces linezolid (57, 58). Thus, the activity of linezolid against biofilm-associated infections remains a topic of controversy (52, 59–61). In staphylococcal biofilms, tolerance to different antibiotics has been linked to the presence of eDNA (22, 23, 62). Specifically, eDNA provides structural integrity limiting antibiotic diffusion and penetration through staphylococcal biofilm (22, 23, 63, 64). In this study, we used the BioFilm Ring Test to quantify the contribution of eDNA in the early stages of biofilm formation. The results showed that DNase effectively prevented biofilm formation, leading to a significant reduction of 26.0 ± 20.4% in the initial attachment of MRSA strains compared to that of untreated control strains. In addition, eDNA was detected in all biofilm samples at a median concentration of 0.95 ng/μL. Notably, the high content of eDNA within the biofilm matrix was significantly correlated with increased tolerance to dalbavancin and vancomycin but not to linezolid. These data suggested that eDNA directly affects the antimicrobial potential of glycopeptides through the biofilm but does not interfere with linezolid activity. Similar conclusions were previously observed for vancomycin, describing an eDNA-based mechanism of antibiotic tolerance (22, 65). Dalbavancin and vancomycin are positively charged under physiological conditions, suggesting that negatively charged eDNA could bind to and interact with those antibiotics (66, 67). Our data showed that exogenous DNA could increase the bacterial counts in biofilm cultures exposed for 20 h to the MBEC_90_ of dalbavancin. Overall, our findings are in agreement with earlier reports showing that S. aureus can incorporate heterologous DNA into the biofilm matrix from an exogenous source, including salmon sperm DNA, thus providing additional evidence that eDNA in the biofilm matrix contributes to increased biofilm tolerance to dalbavancin (22, 23, 68). The exposure of S. epidermidis biofilms to sub-MICs of vancomycin was enriched in eDNA (22, 69). Others described that subinhibitory concentrations of clindamycin increased the ability of S. aureus to form biofilms, shifting the biofilm matrix’s composition toward higher eDNA content (70). The relative eDNA abundance in the biofilm matrix increased after 20 h of exposure to MBEC_90_ but not at sub-MIC_90_ levels of dalbavancin. These results suggest that the biofilm exposure to dalbavancin at MBEC_90_ increases the level of eDNA in the biofilm culture, which in turn, shields MRSA cells from the action of dalbavancin. What remains uncertain is how the relative abundance of eDNA accumulates in the biofilm after dalbavancin treatment. Lysis-independent eDNA release was described for Enterococcus faecalis, Bacillus subtilis, and Neisseria gonorrhoeae (71–74). In *ica*-independent MSSA strains, some genes are important for the eDNA release in the biofilm matrix. A possible mechanism is correlated with the selective lysis of a subset of cells that leads to the release of genomic DNA into the matrix (62, 75, 76). From our study, it seems most reasonable to assume that eDNA accumulates due to the lysis of a subpopulation of cells instead of as a result of an active release into the biofilm matrix dependent on antibiotic exposure. Indeed, at sub-MIC_90_ dalbavancin, we could not observe any significant increase in the relative abundance of eDNA compared to untreated controls.

From a clinical point of view, our study suffers from some limitations. First, our analysis was performed on MRSA strains. Previous studies have shown that S. aureus produces biofilms through two major pathways, alternatively leading to the assembly of a polysaccharide-based or an eDNA/protein biofilm. Although these distinctive forms of biofilms are not mutually exclusive, the polysaccharide-based biofilm is predominantly described in methicillin-sensitive S. aureus (MSSA) strains, while the eDNA/protein biofilm is more represented in MRSA strains (13–16). Consequently, the activity of different antibiotics observed in this study has to be related to an MRSA strain which forms biofilms where eDNA plays a relevant contribution in shaping the architecture of the exopolysaccharide (EPS) matrix (75, 77). Second, in this study, bacteria were exposed to constant concentrations of antibiotics for a prolonged period. These conditions do not mimic the pharmacokinetic profile of the drugs at the site of infection; thus, the clinical findings should be interpreted with caution.

In conclusion, our data show that dalbavancin, at concentrations achievable in human serum, has antimicrobial potential against established MRSA biofilms, representing a promising therapeutic option for treating biofilm-associated SSTI. Furthermore, the evidence that the eDNA can reduce drug-induced antimicrobial activity may offer novel insight for designing more targeted therapeutic strategies against MRSA to either prevent or eradicate harmful biofilms.

## MATERIALS AND METHODS

From the Microbial Strain Repository of the laboratory of Clinical Pathology and Microbiology (San Gallicano Dermatology Institute, Rome, Italy), 32 MRSA strains, collected during the period 2018 to 2020, from 32 patients presenting with complicated SSTIs were included in the study (78). Sample collection, bacterial identification, and antimicrobial susceptibility testing were performed as previously described (79). Strains were classified as MRSA when presenting the gene for methicillin resistance (*mecA*), oxacillin resistance (MIC ≥ 4 mg/mL), and positive agglutination test for penicillin-binding protein (PBP2; Oxoid, Basingstoke, UK) (80).

The ethics committee I.R.C.C.S. Lazio approved the study (Protocol 4394—31.03.2020, trials registry no. 1326/20).

### Biofilm production.

Biofilm production was quantified with the clinical BioFilm Ring Test (cBRT) as previously described (81), using the reagents and equipment provided by the Biofilm Ring Test kit (KITC004), and analyzed with BFC Elements 3.0 software (Biofilm Control, Saint Beauzire, France). S. aureus strain ATCC 25923 and Staphylococcus epidermidis ATCC 12228 (Se12228) were included in each plate as the standard reference and internal control, respectively. Each strain was analyzed in duplicate, and experiments were repeated three times.

### Susceptibility testing.

**MIC.** MICs were determined for each strain using the broth microdilution method, and results were interpreted according to the European Committee on Antimicrobial Susceptibility Testing (EUCAST) clinical breakpoints (http://www.eucast.org/clinical_breakpoints). After the antibiotic treatment, viable cells were determined by plate counting for the CFU/mL determination. A standard bacterial inoculum of 5 × 10^5^ CFU/mL was used. Serial 2-fold dilutions of the antimicrobials were prepared in cation-adjusted Mueller-Hinton broth (MHB). The MIC_90_ was defined as the lowest concentration of antibiotic that killed 90% of the bacteria compared to the untreated control. Experiments were conducted in triplicate.

**Minimum biofilm eradication concentration (MBEC) assays.** For each experiment, an overnight culture of MRSA grown on a blood agar plate was used to inoculate 2 mL of 0.45% saline solution to 0.5 ± 0.1 McFarland turbidity standard (approximately 10^8^ CFU/mL). For biofilm cultures, diluted cell suspensions (approximately 10^5^ CFU/mL) were used to inoculate a 96-well polystyrene flat-bottom plate with 100 μL MHB. After 5 h at 37°C, the wells were rinsed with 0.45% saline solution to remove nonadherent bacteria, and the cells were resuspended in 100 μL of MHB supplemented with serial dilutions of dalbavancin, linezolid, and vancomycin. The plate was incubated for 20 additional hours at 37°C. After 20 h of exposure, the well contents were aspirated. Each well was washed two times with sterile deionized water, and the cells were resuspended in 100 μL of MHB. Biofilms were scraped thoroughly, and the total number of viable cells was determined by serial dilution and plating on blood agar plates to estimate the CFU number. To allow reproducibility in the cell counting procedures, the S. aureus strain ATCC 25923 was included in each plate as the standard reference and internal control. The MBEC_90_ levels were determined as the lower concentrations of antibiotics that killed 90% of the bacteria in preformed biofilms compared to the untreated control.

### Determination of metabolic activity.

The metabolic activity of planktonic and biofilm MRSA isolates was determined using a resazurin-based assay as previously described (82). An overnight culture of MRSA grown on a blood agar plate was used to inoculate 2 mL of 0.45% saline solution to 0.5 ± 0.1 McFarland turbidity standard (approximately 10^8^ CFU/mL). For planktonic cultures, diluted cell suspensions (approximately 10^5^ CFU/mL) were used to inoculate a 96-well polystyrene flat-bottom plate with 100 μL of an MHB/resazurin solution (Promega, Madison, WI, USA). The plates were incubated for 24 h at 37°C, and absorbance (570 nm) was recorded in 20-min periods for 1,200 min using a multidetection microplate reader (Multiskan SkyHigh; Thermo Fisher Scientific, USA).

For biofilm formation, 100 μL of diluted cells suspensions (approximately 10^5^ CFU/mL) in MHB was transferred to a 96-well polystyrene flat-bottom plate. After 5 h at 37°C, the wells were rinsed with 0.45% saline solution, and 100 μL of an MHB/resazurin solution (Promega, Madison, WI, USA) was added. The plate was incubated for 20 additional hours at 37°C, and absorbance (570 nm) was recorded in 20-min periods for 1,200 min using a multidetection microplate reader (Multiskan SkyHigh; Thermo Fisher Scientific, USA).

### Assessment of MRSA biofilm composition.

The Biofilm Ring Test method was used to quantify the attachment and initial biofilm formation in the presence of DNase (100 μg/mL) (83). Standardized bacterial suspensions containing 1 vol % magnetic beads were supplemented with DNase (100 μg/mL) and incubated at 37°C in a 96-well microplate (200 μL/well) (BD Falcon 96 flat-bottom transparent; Corning, USA). Negative controls contained 200 μL of sterile brain heart infusion (BHI) with magnetic beads and enzymes. The plate was read after 6 h of incubation, as described above. The capacity of the strains to form biofilm in the presence of DNase was expressed using the relative difference (RD):

(1) RD = [(Pmb without enzyme  –  Pmb with enzyme)/Pmb without enzyme] × 100

The analysis was performed three times in duplicate for each sample.

### eDNA quantification in MRSA biofilm.

eDNA was quantified as described previously (84). Briefly, a microtiter plate was inoculated with diluted starter cultures adjusted to a final concentration of approximately 1 × 10^5^ CFU/mL in 100 μL of MHB and incubated at room temperature under static conditions for 20 h. The presence of eDNA was quantified by the addition of 100 μL Tris-EDTA (TE) buffer followed by 100 μL freshly made PicoGreen solution (1 μL PicoGreen dye in 199 μL TE buffer). Wells with PicoGreen were incubated for 5 min before measuring the fluorescence intensity (excitation 485 nm/emission 535 nm, 0.1 s) using a fluorescence plate reader (Wallace Victor 3, 1420 Multicolor; PerkinElmer). Each analysis was performed on three biological replicates for each strain. Lambda DNA (Invitrogen Molecular Probes) was used to generate a standard curve for each run. For each time point, the biofilm was stained with PicoGreen and observed using phase-contrast and fluorescence microscopy (Zeiss Axiovert 200M).

To test the effects of exogenous DNA on the growth of MRSA biofilm, MHB was supplemented with dalbavancin, salmon sperm DNA (16 μg/mL) alone, or both dalbavancin and salmon sperm DNA. Dalbavancin and salmon sperm DNA were preincubated at 25°C for 30 min before cells were added. For biofilm formation, 100 μL of diluted cell suspensions (approximately 10^5^ CFU/mL) in MHB was transferred to a 96-well polystyrene flat-bottom plate. After 5 h at 37°C, wells were rinsed with 0.45% saline solution, and 100 μL of salmon sperm DNA (16 μg/mL) and dalbavancin, separately and in combination, were added. After 20 h of incubation at 37°C, the biofilms were washed thoroughly with sterile deionized water. Biofilms were scraped, and the total number of viable cells was determined by serial dilution and plating on blood agar plates to estimate the CFU number.

### Biofilm imaging.

Biofilms were grown in μ-Slide slides (Ibidi, Gräfelfing, Germany) inoculated with ∼1 × 10^5^ cells in 500 μL of fresh BHI medium and incubated for 48 h at 37°C. The culture medium was changed after 24 h of biofilm growth. Biofilms were stained using the Live/Dead BacLight bacterial viability kit (Life Technologies, New York, NY, USA) and/or TOTO-1 iodide staining (Thermo Fisher Scientific, catalog [cat.] no. T3600; dilution, 1:1,000) for detection of free eDNA surrounding living and dead cells (85, 86) and examined with an Apotome system (Zeiss, Oberkochen, Germany) connected to an Axio Observer inverted fluorescence microscope (Zeiss). Data were analyzed with the ZEN 3.1 (blue edition) software (Zeiss).

### Statistics.

All variables were summarized with descriptive statistics and tested for normality. When appropriate, comparisons between continuous variables were carried out with Student’s *t* test or the Mann-Whitney U test. In contrast, when appropriate, categorical variables were tested using the χ^2^ or two-tailed Fisher’s exact test. Correlation analysis was performed using the Spearman rank-order correlation coefficient (ρ). A *P* value of <0.05 was considered statistically significant. Statistical analyses were performed using SPSS software version 21 (SPSS, Inc., Chicago, IL, USA).

## References

[1] Leong HN, Kurup A, Tan MY, Kwa ALH, Liau KH, Wilcox MH. 2018. Management of complicated skin and soft tissue infections with a special focus on the role of newer antibiotics. Infect Drug Resist 11:1959–1974. doi:10.2147/IDR.S172366. 30464538 PMC6208867

[2] Manian FA. 2014. The role of postoperative factors in surgical site infections: time to take notice. Clin Infect Dis 59:1272–1276. doi:10.1093/cid/ciu552. 25028464

[3] King MD, Humphrey BJ, Wang YF, Kourbatova EV, Ray SM, Blumberg HM. 2006. Emergence of community-acquired methicillin-resistant Staphylococcus aureus USA 300 clone as the predominant cause of skin and soft-tissue infections. Ann Intern Med 144:309–317. doi:10.7326/0003-4819-144-5-200603070-00005. 16520471

[4] Zarb P, Coignard B, Griskeviciene J, Muller A, Vankerckhoven V, Weist K, Goossens M, Vaerenberg S, Hopkins S, Catry B, Monnet D, Goossens H, Suetens C. 2012. National Contact Points for the ECDC pilot point prevalence survey, Hospital Contact Points for the ECDC pilot point prevalence survey. The European Centre for Disease Prevention and Control (ECDC) pilot point prevalence survey of healthcare-associated infections and antimicrobial use. Euro Surveill 17:20316. doi:10.2807/ese.17.46.20316-en. 23171822

[5] Ray GT, Suaya JA, Baxter R. 2013. Incidence, microbiology, and patient characteristics of skin and soft-tissue infections in a U.S. population: a retrospective population-based study. BMC Infect Dis 13:252. doi:10.1186/1471-2334-13-252. 23721377 PMC3679727

[6] Dryden M, Andrasevic AT, Bassetti M, Bouza E, Chastre J, Baguneid M, Esposito S, Giamarellou H, Gyssens I, Nathwani D, Unal S, Voss A, Wilcox M. 2015. Managing skin and soft-tissue infection and nosocomial pneumonia caused by MRSA: a 2014 follow-up survey. Int J Antimicrob Agents 45:S1–S14. doi:10.1016/S0924-8579(15)30002-9. 25867210

[7] Loewen K, Schreiber Y, Kirlew M, Bocking N, Kelly L. 2017. Community-associated methicillin-resistant Staphylococcus aureus infection: literature review and clinical update. Can Fam Physician 63:512–520. 28701438 PMC5507223

[8] Mellinghoff SC, Vehreschild JJ, Liss BJ, Cornely OA. 2019. Metadata correction: epidemiology of surgical site infections with Staphylococcus aureus in Europe: protocol for a retrospective, multicenter study. JMIR Res Protoc 8:e10712. doi:10.2196/10712. 30617045 PMC6329409

[9] Kawamura H, Nishi J, Imuta N, Tokuda K, Miyanohara H, Hashiguchi T, Zenmyo M, Yamamoto T, Ijiri K, Kawano Y, Komiya S. 2011. Quantitative analysis of biofilm formation of methicillin-resistant Staphylococcus aureus (MRSA) strains from patients with orthopaedic device-related infections. FEMS Immunol Med Microbiol 63:10–15. doi:10.1111/j.1574-695X.2011.00821.x. 21595755

[10] Lebeaux D, Ghigo JM, Beloin C. 2014. Biofilm-related infections: bridging the gap between clinical management and fundamental aspects of recalcitrance toward antibiotics. Microbiol Mol Biol Rev 78:510–543. doi:10.1128/MMBR.00013-14. 25184564 PMC4187679

[11] Di Domenico EG, Farulla I, Prignano G, Gallo MT, Vespaziani M, Cavallo I, Sperduti I, Pontone M, Bordignon V, Cilli L, De Santis A, Di Salvo F, Pimpinelli F, Lesnoni La Parola I, Toma L, Ensoli F. 2017. Biofilm is a major virulence determinant in bacterial colonization of chronic skin ulcers independently from the multidrug resistant phenotype. Int J Mol Sci 18:1077. doi:10.3390/ijms18051077. 28513576 PMC5454986

[12] Nguyen HTT, Nguyen TH, Otto M. 2020. The staphylococcal exopolysaccharide PIA - Biosynthesis and role in biofilm formation, colonization, and infection. Comput Struct Biotechnol J 18:3324–3334. doi:10.1016/j.csbj.2020.10.027. 33240473 PMC7674160

[13] Mlynek KD, Bulock LL, Stone CJ, Curran LJ, Sadykov MR, Bayles KW, Brinsmade SR. 2020. Genetic and biochemical analysis of CodY-mediated cell aggregation in Staphylococcus aureus reveals an interaction between extracellular DNA and polysaccharide in the extracellular matrix. J Bacteriol 202:e00593-19. doi:10.1128/JB.00593-19. 32015143 PMC7099133

[14] Fitzpatrick F, Humphreys H, O'Gara JP. 2005. Evidence for icaADBC-independent biofilm development mechanism in methicillin-resistant Staphylococcus aureus clinical isolates. J Clin Microbiol 43:1973–1976. doi:10.1128/JCM.43.4.1973-1976.2005. 15815035 PMC1081404

[15] O’Neill E, Pozzi C, Houston P, Humphreys H, Robinson DA, Loughman A, Foster TJ, O’Gara JP. 2008. A novel Staphylococcus aureus biofilm phenotype mediated by the fibronectin-binding proteins, FnBPA and FnBPB. J Bacteriol 190:3835–3850. doi:10.1128/JB.00167-08. 18375547 PMC2395027

[16] McCarthy H, Rudkin JK, Black NS, Gallagher L, O’Neill E, O’Gara JP. 2015. Methicillin resistance and the biofilm phenotype in Staphylococcus aureus. Front Cell Infect Microbiol 5:1. 25674541 10.3389/fcimb.2015.00001 PMC4309206

[17] Seltzer E, Dorr MB, Goldstein BP, Perry M, Dowell JA, Henkel T, Dalbavancin Skin and Soft-Tissue Infection Study Group. 2003. Once-weekly dalbavancin versus standard-of-care antimicrobial regimens for treatment of skin and soft-tissue infections. Clin Infect Dis 37:1298–1303. doi:10.1086/379015. 14583862

[18] Baldoni D, Furustrand Tafin U, Aeppli S, Angevaare E, Oliva A, Haschke M, Zimmerli W, Trampuz A. 2013. Activity of dalbavancin, alone and in combination with rifampicin, against meticillin-resistant Staphylococcus aureus in a foreign-body infection model. Int J Antimicrob Agents 42:220–225. doi:10.1016/j.ijantimicag.2013.05.019. 23880168

[19] Rubia M, Cordero A, Pérez-Granda MJ, Cercenado E, Pascual C, Muñoz P, Guembe M. 2020. In vitro study to evaluate the bioactivity of freezing a heparin-based dalbavancin lock solution. Antimicrob Agents Chemother 64:e01495-20. doi:10.1128/AAC.01495-20. PMC7674050 32988823

[20] Díaz-Ruíz C, Alonso B, Cercenado E, Cruces R, Bouza E, Muñoz P, Guembe M. 2018. Can dalbavancin be used as a catheter lock solution? J Med Microbiol 67:936–944. doi:10.1099/jmm.0.000749. 29771236

[21] Kussmann M, Obermueller M, Berndl F, Reischer V, Veletzky L, Burgmann H, Poeppl W, 2018. Dalbavancin for treatment of implant-related methicillin-resistant Staphylococcus aureus osteomyelitis in an experimental rat model. Sci Rep 8:9661–9682. doi:10.1038/s41598-018-28006-8. 29941909 PMC6018549

[22] Dengler V, Foulston L, DeFrancesco AS, Losick R. 2015. An electrostatic net model for the role of extracellular DNA in biofilm formation by Staphylococcus aureus. J Bacteriol 197:3779–3787. doi:10.1128/JB.00726-15. 26416831 PMC4652055

[23] Agarwal R, Bartsch SM, Kelly BJ, Prewitt M, Liu Y, Chen Y, Umscheid CA. 2018. Newer glycopeptide antibiotics for treatment of complicated skin and soft tissue infections: systematic review, network meta-analysis and cost analysis. Clin Microbiol Infect 24:361–368. doi:10.1016/j.cmi.2017.08.028. 28882727 PMC5925741

[24] Pfaller MA, Mendes RE, Duncan LR, Flamm RK, Sader HS. 2018. Activity of dalbavancin and comparator agents against Gram-positive cocci from clinical infections in the USA and Europe 2015–2016. J Antimicrob Chemother 73:2748–2756. doi:10.1093/jac/dky235. 29982565

[25] Esposito S, Bassetti M, Borre S, Bouza E, Dryden M, Fantoni M, Gould IM, Leoncini F, Leone S, Milkovich G, Nathwani D, Segreti J, Sganga G, Unal S, Venditti M, International Society of Chemotherapy, Italian Society of Infectious Tropical Diseases. 2011. Diagnosis and management of skin and soft-tissue infections (SSTI): a literature review and consensus statement on behalf of the Italian Society of Infectious Diseases and International Society of Chemotherapy. J Chemother 23:251–262. doi:10.1179/joc.2011.23.5.251. 22005055

[26] Bounthavong M, Hsu DI. 2010. Efficacy and safety of linezolid in methicillin-resistant Staphylococcus aureus (MRSA) complicated skin and soft tissue infection (cSSTI): a meta-analysis. Curr Med Res Opin 26:407–421. doi:10.1185/03007990903454912. 20001574

[27] Stevens DL, Herr D, Lampiris H, Hunt JL, Batts DH, Hafkin B, Linezolid MRSA Study Group. 2002. Linezolid versus vancomycin for the treatment of methicillin-resistant Staphylococcus aureus infections. Clin Infect Dis 34:1481–1490. doi:10.1086/340353. 12015695

[28] James GA, Swogger E, Wolcott R, Pulcini E, Secor P, Sestrich J, Costerton JW, Stewart PS. 2008. Biofilms in chronic wounds. Wound Repair Regen 16:37–44. doi:10.1111/j.1524-475X.2007.00321.x. 18086294

[29] Rahim K, Saleha S, Zhu X, Huo L, Basit A, Franco OL. 2017. Bacterial contribution in chronicity of wounds. Microb Ecol 73:710–721. doi:10.1007/s00248-016-0867-9. 27742997

[30] Kiedrowski MR, Horswill AR. 2011. New approaches for treating staphylococcal biofilm infections. Ann N Y Acad Sci 1241:104–121. doi:10.1111/j.1749-6632.2011.06281.x. 22191529

[31] Merritt C, Haran JP, Mintzer J, Stricker J, Merchant RC. 2013. All purulence is local: epidemiology and management of skin and soft tissue infections in three urban emergency departments. BMC Emerg Med 13:26. doi:10.1186/1471-227X-13-26. 24359038 PMC3878171

[32] Karamatsu ML, Thorp AW, Brown L. 2012. Changes in community-associated methicillin-resistant Staphylococcus aureus skin and soft tissue infections presenting to the pediatric emergency department: comparing 2003 to 2008. Pediatr Emerg Care 28:131–135. doi:10.1097/PEC.0b013e318243fa36. 22270497

[33] Otto M. 2018. Staphylococcal biofilms. Microbiol Spectr 6:6.4.27. doi:10.1128/microbiolspec.GPP3-0023-2018. PMC6282163 30117414

[34] Pulcrano G, Vollaro A, Rossano F, Catania MR. 2013. Molecular and phenotypic characterization of methicillin-resistant Staphylococcus aureus from surgical site infections. Surg Infect (Larchmt) 14:196–202. doi:10.1089/sur.2012.002. 23530808

[35] Kwiecinski J, Kahlmeter G, Jin T. 2015. Biofilm formation by Staphylococcus aureus isolates from skin and soft tissue infections. Curr Microbiol 70:698–703. doi:10.1007/s00284-014-0770-x. 25586078

[36] Khalili H, Najar-Peerayeh S, Mahrooghi M, Mansouri P, Bakhshi B. 2021. Methicillin-resistant Staphylococcus aureus colonization of infectious and non-infectious skin and soft tissue lesions in patients in Tehran. BMC Microbiol 21:282. doi:10.1186/s12866-021-02340-w. 34657594 PMC8521987

[37] Di Domenico EG, Cavallo I, Capitanio B, Ascenzioni F, Pimpinelli F, Morrone A, Ensoli F. 2019. Staphylococcus aureus and the cutaneous microbiota biofilms in the pathogenesis of atopic dermatitis. Microorganisms 7:301. doi:10.3390/microorganisms7090301. 31470558 PMC6780378

[38] Shin K, Yun Y, Yi S, Lee HG, Cho JC, Suh KD, Lee J, Park J. 2013. Biofilm-forming ability of Staphylococcus aureus strains isolated from human skin. J Dermatol Sci 71:130–137. doi:10.1016/j.jdermsci.2013.04.004. 23664186

[39] Choo EJ, Chambers HF. 2016. Treatment of methicillin-resistant Staphylococcus aureus bacteremia. Infect Chemother 48:267–273. doi:10.3947/ic.2016.48.4.267. 28032484 PMC5204005

[40] Žiemytė M, Rodríguez-Díaz JC, Ventero MP, Mira A, Ferrer MD. 2020. Effect of dalbavancin on staphylococcal biofilms when administered alone or in combination with biofilm-detaching compounds. Front Microbiol 11:553. doi:10.3389/fmicb.2020.00553. 32362877 PMC7180179

[41] Di Pilato V, Ceccherini F, Sennati S, D’Agostino F, Arena F, D’Atanasio N, Di Giorgio FP, Tongiani S, Pallecchi L, Rossolini GM. 2020. In vitro time-kill kinetics of dalbavancin against Staphylococcus spp. biofilms over prolonged exposure times. Diagn Microbiol Infect Dis 96:114901. doi:10.1016/j.diagmicrobio.2019.114901. 31761480

[42] Fernández J, Greenwood-Quaintance KE, Patel R. 2016. In vitro activity of dalbavancin against biofilms of staphylococci isolated from prosthetic joint infections. Diagn Microbiol Infect Dis 85:449–451. doi:10.1016/j.diagmicrobio.2016.05.009. 27241369

[43] Darouiche RO, Mansouri MD. 2005. Dalbavancin compared with vancomycin for prevention of Staphylococcus aureus colonization of devices in vivo. J Infect 50:206–209. doi:10.1016/j.jinf.2004.05.006. 15780414

[44] Oliva A, Stefani S, Venditti M, Di Domenico EG. 2021. Biofilm-related infections in Gram-positive bacteria and the potential role of the long-acting agent dalbavancin. Front Microbiol 12:749685. doi:10.3389/fmicb.2021.749685. 34745053 PMC8569946

[45] Barnea Y, Lerner A, Aizic A, Navon-Venezia S, Rachi E, Dunne MW, Puttagunta S, Carmeli Y. 2016. Efficacy of dalbavancin in the treatment of MRSA rat sternal osteomyelitis with mediastinitis. J Antimicrob Chemother 71:460–463. doi:10.1093/jac/dkv357. 26518048

[46] Chen M, Yu Q, Sun H. 2013. Novel strategies for the prevention and treatment of biofilm-related infections. Int J Mol Sci 14:18488–18501. doi:10.3390/ijms140918488. 24018891 PMC3794791

[47] Streit JM, Fritsche TR, Sader HS, Jones RN. 2004. Worldwide assessment of dalbavancin activity and spectrum against over 6,000 clinical isolates. Diagn Microbiol Infect Dis 48:137–143. doi:10.1016/j.diagmicrobio.2003.09.004. 14972384

[48] Jefferson KK, Goldmann DA, Pier GB. 2005. Use of confocal microscopy to analyze the rate of vancomycin penetration through Staphylococcus aureus biofilms. Antimicrob Agents Chemother 49:2467–2473. doi:10.1128/AAC.49.6.2467-2473.2005. 15917548 PMC1140491

[49] Kaneko H, Nakaminami H, Ozawa K, Wajima T, Noguchi N. 2021. In vitro anti-biofilm effect of anti-methicillin-resistant Staphylococcus aureus (anti-MRSA) agents against the USA300 clone. J Glob Antimicrob Resist 24:63–71. doi:10.1016/j.jgar.2020.11.026. 33307275

[50] Swaney SM, Aoki H, Ganoza MC, Shinabarger DL. 1998. The oxazolidinone linezolid inhibits initiation of protein synthesis in bacteria. Antimicrob Agents Chemother 42:3251–3255. doi:10.1128/AAC.42.12.3251. 9835522 PMC106030

[51] Smith K, Perez A, Ramage G, Gemmell CG, Lang S. 2009. Comparison of biofilm-associated cell survival following in vitro exposure of meticillin-resistant Staphylococcus aureus biofilms to the antibiotics clindamycin, daptomycin, linezolid, tigecycline and vancomycin. Int J Antimicrob Agents 33:374–378. doi:10.1016/j.ijantimicag.2008.08.029. 19101124

[52] Giacometti A, Cirioni O, Ghiselli R, Orlando F, Mocchegiani F, Silvestri C, Licci A, De Fusco M, Provinciali M, Saba V, Scalise G. 2005. Comparative efficacies of quinupristin-dalfopristin, linezolid, vancomycin, and ciprofloxacin in treatment, using the antibiotic-lock technique, of experimental catheter-related infection due to Staphylococcus aureus. Antimicrob Agents Chemother 49:4042–4045. doi:10.1128/AAC.49.10.4042-4045.2005. 16189078 PMC1251555

[53] Wu S, Yang T, Luo Y, Li X, Zhang X, Tang J, Ma X, Wang Z. 2014. Efficacy of the novel oxazolidinone compound FYL-67 for preventing biofilm formation by Staphylococcus aureus. J Antimicrob Chemother 69:3011–3019. doi:10.1093/jac/dku240. 24997316

[54] Fernandez-Hidalgo N, Gavalda J, Almirante B, Martín MT, Onrubia PL, Gomis X, Pahissa A. 2010. Evaluation of linezolid, vancomycin, gentamicin and ciprofloxacin in a rabbit model of antibiotic-lock technique for Staphylococcus aureus catheter-related infection. J Antimicrob Chemother 65:525–530. doi:10.1093/jac/dkp499. 20083550

[55] El-Azizi M, Rao S, Kanchanapoom T, Khardori N. 2005. In vitro activity of vancomycin, quinupristin/dalfopristin, and linezolid against intact and disrupted biofilms of staphylococci. Ann Clin Microbiol Antimicrob 4:2. doi:10.1186/1476-0711-4-2. 15638934 PMC546415

[56] Yue J, Dong BR, Yang M, Chen X, Wu T, Liu GJ. 2016. Linezolid versus vancomycin for skin and soft tissue infections. Cochrane Database Syst Rev 7:CD008056. doi:10.1002/14651858.CD008056.pub3. PMC10435313 26758498

[57] Beibei L, Yun C, Mengli C, Nan B, Xuhong Y, Rui W. 2010. Linezolid versus vancomycin for the treatment of Gram‐positive bacterial infections: meta‐analysis of randomised controlled trials. Int J Antimicrob Agents 35:3–12. doi:10.1016/j.ijantimicag.2009.09.013. 19900794

[58] Raad I, Hanna H, Jiang Y, Dvorak T, Reitzel R, Chaiban G, Sherertz R, Hachem R. 2007. Comparative activities of daptomycin, linezolid, and tigecycline against catheter-related methicillin-resistant Staphylococcus bacteremic isolates embedded in biofilm. Antimicrob Agents Chemother 51:1656–1660. doi:10.1128/AAC.00350-06. 17353249 PMC1855569

[59] Cafiso V, Bertuccio T, Spina D, Purrello S, Stefani S. 2010. Tigecycline inhibition of a mature biofilm in clinical isolates of Staphylococcus aureus: comparison with other drugs. FEMS Immunol Med Microbiol 59:466–469. doi:10.1111/j.1574-695X.2010.00701.x. 20528931

[60] Abad L, Tafani V, Tasse J, Josse J, Chidiac C, Lustig S, Ferry T, Diot A, Laurent F, Valour F. 2019. Evaluation of the ability of linezolid and tedizolid to eradicate intraosteoblastic and biofilm-embedded Staphylococcus aureus in the bone and joint infection setting. J Antimicrob Chemother 74:625–632. doi:10.1093/jac/dky473. 30517641

[61] Bose JL, Lehman MK, Fey PD, Bayles KW. 2012. Contribution of the Staphylococcus aureus Atl AM and GL murein hydrolase activities in cell division, autolysis, and biofilm formation. PLoS One 7:e42244. doi:10.1371/journal.pone.0042244. 22860095 PMC3409170

[62] Okshevsky M, Meyer RL. 2015. The role of extracellular DNA in the establishment, maintenance and perpetuation of bacterial biofilms. Crit Rev Microbiol 41:341–352. doi:10.3109/1040841X.2013.841639. 24303798

[63] Sugimoto S, Sato F, Miyakawa R, Chiba A, Onodera S, Hori S, Mizunoe Y. 2018. Broad impact of extracellular DNA on biofilm formation by clinically isolated methicillin-resistant and -sensitive strains of Staphylococcus aureus. Sci Rep 8:2254. doi:10.1038/s41598-018-20485-z. 29396526 PMC5797107

[64] Daddi Oubekka S, Briandet R, Fontaine-Aupart M-P, Steenkeste K. 2012. Correlative time-resolved fluorescence microscopy to assess antibiotic diffusion-reaction in biofilms. Antimicrob Agents Chemother 56:3349–3358. doi:10.1128/AAC.00216-12. 22450986 PMC3370788

[65] Economou NJ, Nahoum V, Week SD, Grasty KCI, Zentner J, Townsend TM, Bhuiya MW, Cocklin S, Loll PJ. 2012. A carrier protein strategy yields the structure of dalbavancin. J Am Chem Soc 134:4637–4645. doi:10.1021/ja208755j. 22352468 PMC3304006

[66] Johnson JL, Yalkowsky SH. 2006. Reformulation of a new vancomycin analog: an example of the importance of buffer species and strength. AAPS PharmSciTech 7:E33–E37. doi:10.1208/pt070105.28290020

[67] Foulston L, Elsholz AK, DeFrancesco AS, Losick R. 2014. The extracellular matrix of Staphylococcus aureus biofilms comprises cytoplasmic proteins that associate with the cell surface in response to decreasing pH. mBio 5:e01667-14. doi:10.1128/mBio.01667-14. 25182325 PMC4173787

[68] Kaplan JB, Jabbouri S, Sadovskaya I. 2011. Extracellular DNA dependent biofilm formation by Staphylococcus epidermidis RP62A in response to subminimal inhibitory concentrations of antibiotics. Res Microbiol 162:535–541. doi:10.1016/j.resmic.2011.03.008. 21402153 PMC3109171

[69] Doroshenko N, Tseng BS, Howlin RP, Deacon J, Wharton JA, Thurner PJ, Gilmore BF, Parsek MR, Stoodley P. 2014. Extracellular DNA impedes the transport of vancomycin in Staphylococcus epidermidis biofilms preexposed to subinhibitory concentrations of vancomycin. Antimicrob Agents Chemother 58 7273. doi:10.1128/AAC.03132-14. 25267673 PMC4249571

[70] Barnes AMT, Ballering KS, Leibman RS, Wells CL, Dunny GM. 2012. Enterococcus faecalis produces abundant extracellular structures containing DNA in the absence of cell lysis during early biofilm formation. mBio 3:e00193-12. doi:10.1128/mBio.00193-12. 22829679 PMC3413405

[71] Zafra O, Lamprecht-Grandío M, de Figueras CG, González-Pastor JE. 2012. Extracellular DNA release by undomesticated Bacillus subtilis is regulated by early competence. PLoS One 7:e48716. doi:10.1371/journal.pone.0048716. 23133654 PMC3487849

[72] Zweig M, Schork S, Koerdt A, Siewering K, Sternberg C, Thormann K, Albers SV, Molin S, van der Does C. 2014. Secreted single-stranded DNA is involved in the initial phase of biofilm formation by Neisseria gonorrhoeae. Environ Microbiol 16:1040–1052. doi:10.1111/1462-2920.12291. 24119133

[73] Jakubovics NS, Shields RC, Rajarajan N, Burgess JG. 2013. Life after death: the critical role of extracellular DNA in microbial biofilms. Lett Appl Microbiol 57:467–475. doi:10.1111/lam.12134. 23848166

[74] DeFrancesco AS, Masloboeva N, Syed AK, DeLoughery A, Bradshaw N, Li GW, Gilmore MS, Walker S, Losick R. 2017. Genome-wide screen for genes involved in eDNA release during biofilm formation by Staphylococcus aureus. Proc Natl Acad Sci USA 114:E5969–E5978. doi:10.1073/pnas.1704544114. 28674000 PMC5530685

[75] Boles BR, Thoendel M, Roth AJ, Horswill AR. 2010. Identification of genes involved in polysaccharide-independent Staphylococcus aureus biofilm formation. PLoS One 5:e10146. doi:10.1371/journal.pone.0010146. 20418950 PMC2854687

[76] Rice KC, Mann EE, Endres JL, Weiss EC, Cassat JE, Smeltzer MS, Bayles KW. 2007. The cidA murein hydrolase regulator contributes to DNA release and biofilm development in Staphylococcus aureus. Proc Natl Acad Sci USA 104:8113–8118. doi:10.1073/pnas.0610226104. 17452642 PMC1876580

[77] Bassetti M, Baguneid M, Bouza E, Dryden M, Nathwani D, Wilcox M. 2014. European perspective and update on the management of complicated skin and soft tissue infections due to methicillin-resistant Staphylococcus aureus after more than 10 years of experience with linezolid. Clin Microbiol Infect 20:3–18. doi:10.1111/1469-0691.12463. 24580738

[78] Di Domenico EG, Toma L, Prignano G, Pelagalli L, Police A, Cavallotti C, Torelli R, Sanguinetti M, Ensoli F. 2015. Misidentification of Streptococcus uberis as a human pathogen: a case report and literature review. Int J Infect Dis 33:79–81. doi:10.1016/j.ijid.2015.01.002. 25578263

[79] Di Domenico EG, Cavallo I, Bordignon V, Prignano G, Sperduti I, Gurtner A, Trento E, Toma L, Pimpinelli F, Capitanio B, Ensoli F. 2018. Inflammatory cytokines and biofilm production sustain Staphylococcus aureus outgrowth and persistence: a pivotal interplay in the pathogenesis of atopic dermatitis. Sci Rep 8:9573. doi:10.1038/s41598-018-27421-1. 29955077 PMC6023932

[80] Di Domenico EG, Marchesi F, Cavallo I, Toma L, Sivori F, Papa E, Spadea A, Cafarella G, Terrenato I, Prignano G, Pimpinelli F, Mastrofrancesco A, D'Agosto G, Trento E, Morrone A, Mengarelli A, Ensoli F. 2021. The impact of bacterial biofilms on end-organ disease and mortality in patients with hematologic malignancies developing a bloodstream infection. Microbiol Spectr 9:e0055021. doi:10.1128/Spectrum.00550-21. 34406812 PMC8552682

[81] Vandecandelaere I, Van Nieuwerburgh F, Deforce D, Coenye T. 2017. Metabolic activity, urease production, antibiotic resistance and virulence in dual species biofilms of Staphylococcus epidermidis and Staphylococcus aureus. PLoS One 12:e0172700. doi:10.1371/journal.pone.0172700. 28263995 PMC5338783

[82] Tasse J, Trouillet-Assant S, Josse J, Martins-Simões P, Valour F, Langlois-Jacques C, Badel-Berchoux S, Provot C, Bernardi T, Ferry T, Laurent F. 2018. Association between biofilm formation phenotype and clonal lineage in Staphylococcus aureus strains from bone and joint infections. PLoS One 13:e0200064. doi:10.1371/journal.pone.0200064. 30161132 PMC6116976

[83] Tang L, Schramm A, Neu TR, Revsbech NP, Meyer RL. 2013. Extracellular DNA in adhesion and biofilm formation of four environmental isolates: a quantitative study. FEMS Microbiol Ecol 86:394–403. doi:10.1111/1574-6941.12168. 23786537

[84] Turnbull L, Toyofuku M, Hynen AL, Kurosawa M, Pessi G, Petty NK, Osvath SR, Cárcamo-Oyarce G, Gloag ES, Shimoni R, Omasits U, Ito S, Yap X, Monahan LG, Cavaliere R, Ahrens CH, Charles IG, Nomura N, Eberl L, Whitchurch CB. 2016. Explosive cell lysis as a mechanism for the biogenesis of bacterial membrane vesicles and biofilms. Nat Commun 7:11220. doi:10.1038/ncomms11220. 27075392 PMC4834629

[85] Okshevsky M, Meyer RL. 2014. Evaluation of fluorescent stains for visualizing extracellular DNA in biofilms. J Microbiol Methods 105:102–104. doi:10.1016/j.mimet.2014.07.010. 25017901

[86] Di Domenico EG, Cavallo I, Sivori F, Marchesi F, Prignano G, Pimpinelli F, Sperduti I, Pelagalli L, Di Salvo F, Celesti I, Paluzzi S, Pronesti C, Koudriavtseva T, Ascenzioni F, Toma L, De Luca A, Mengarelli A, Ensoli F. 2020. Biofilm production by carbapenem-resistant Klebsiella pneumoniae significantly increases the risk of death in oncological patients. Front Cell Infect Microbiol 10:561741. doi:10.3389/fcimb.2020.561741. 33363047 PMC7759150

